# Varicella-Zoster Myelitis With Thoracic Demyelination and Lumbosacral Plexopathy in the Setting of Uncontrolled HIV

**DOI:** 10.7759/cureus.83689

**Published:** 2025-05-07

**Authors:** Nathan Einhorn, Aditya Grover, Leo Hernandez, Jennifer Marsh

**Affiliations:** 1 Internal Medicine, University of South Florida Morsani College of Medicine, Tampa, USA; 2 Pharmacotherapeutics and Clinical Research, University of South Florida Taneja College of Pharmacy, Tampa, USA

**Keywords:** antiviral therapy, corticosteroids therapy, hiv aids, longitudinally extensive transverse myelitis, varicella myelitis, varicella-zoster virus

## Abstract

Varicella-zoster virus (VZV) myelitis is a rare but serious complication of VZV reactivation that can lead to significant neurological deficits. While antiviral therapy is the mainstay of treatment, the role of corticosteroids remains unclear. We present a case of a 30-year-old male who developed progressive asymmetric lower-extremity weakness, constipation, and urinary retention without a dermatomal rash in the setting of HIV/AIDS. MRI revealed longitudinally extensive transverse myelitis, and cerebrospinal fluid (CSF) analysis confirmed VZV reactivation. The patient was treated with ganciclovir and high-dose corticosteroids, leading to an initial neurological improvement. This case highlights the potential benefit of adjunctive corticosteroid therapy in reducing inflammation and improving outcomes in VZV myelitis. Given the lack of standardized treatment guidelines, further studies are needed to clarify the role of corticosteroids and optimize management strategies for this condition.

## Introduction

The varicella-zoster virus (VZV) is a neurotropic human herpesvirus that is large, enveloped, and has a double-stranded DNA genome [1]. Also known as human herpesvirus 3, VZV targets T lymphocytes, epithelial cells, and ganglia. It causes the primary infection of varicella (chickenpox), followed by latency in ganglionic neurons. As cellular immunity decreases, like with advancing age or immunocompromised states, VZV can reactivate from ganglia and travel anterograde to the skin, resulting in zoster (shingles) [2]. While uncommon, VZV reactivation can also lead to many neurological manifestations, including vasculopathy, meningitis/meningoencephalitis, myelopathy, cerebellitis, and various ocular disorders. VZV myelitis or myelopathy is a rare complication intermittently described in case reports [3]. It usually presents within 1-2 weeks of the zosteriform rash with typical myelitis symptoms of acute leg weakness, sphincter disturbance, or sensory features at the same dermatomal level or with extensive longitudinal spinal cord involvement [4]. Immunocompromised patients tend to have poorer outcomes, and there is a clear temporal relationship between the development of rash and myelopathy [5]. In rarer cases, VZV myelitis may occur without a rash, presenting additional diagnostic challenges [5]. Unfortunately, there are no broadly established treatment guidelines or recommendations for VZV myelitis, which is a significant concern for immunocompromised individuals and the elderly [1]. To elucidate future treatment strategies, we present a case and the corresponding therapy of a patient with VZV myelitis without cutaneous manifestation in the setting of uncontrolled HIV/AIDS.

## Case presentation

We present the case of a 30-year-old male with a past medical history of alcohol use disorder, smoking use disorder, and history of incarceration who presented with 10 days of gradual asymmetric right greater than left leg weakness, numbness, and paresthesia associated with bilateral mid-lower back pain, saddle anesthesia, urinary incontinence, and constipation. The patient also endorsed a decrease in appetite, weight loss, and one-day bilateral blurry, double vision. The patient was seen at an outside hospital three times prior to presentation for these symptoms that resulted in an initial negative workup, including metabolic panel, blood count, inflammatory markers, and CT and MRI of the lumbar spine, with a subsequent diagnosis of an unspecified neurological disease. He was treated with less than one week of pregabalin 150 mg capsule by mouth twice daily, naproxen 500 mg tablet by mouth twice daily, and methylprednisolone with only mild relief. Of note, the patient reported taking only the first dose of methylprednisolone tablets (8 mg total) of a prescribed six-day taper before self-discontinuing. The patient denied any skin rashes, physical trauma, nuchal rigidity, photophobia, history of disc herniation, dysphagia, speech disturbances, sick contacts, fever, muscle fatigue, headaches, history of stroke, myocardial infarction, or cancer.

In the emergency department, the patient was hemodynamically stable and afebrile. On physical examination, he was emaciated with bitemporal wasting. There were no significant dermatologic findings on total body skin exam, including zosteriform rashes. Ocular exam demonstrated left greater than right anisocoria and saccades with intact extraocular movements, visual fields, and pupillary light reflex. The patient was alert and oriented, and cranial nerves II-XII were grossly intact. Spine and back exam had normal range of motion without rigidity, tenderness, spasms, or vertebral step-offs. Bilateral upper extremities and left lower extremity were 5/5 strength to all movements with intact sensation. His right lower extremity was 0/5 strength below the hip without sensation to light touch, temperature, vibration, or proprioception in all nerve distributions. All reflexes were 2+ throughout, the Babinski sign was negative bilaterally, cerebellar tests were within normal limits, and there was normal muscle tone bilaterally. 

Lab workup was significant for elevated erythrocyte sedimentation rate (ESR), decreased absolute CD4 count, elevated HIV PCR, HIV 1 antibody positivity, and a positive VZV PCR and VZV immunoglobulin G (IGG) in serum (Tables 1, 2). Lumbar puncture yielded clear, colorless cerebrospinal fluid (CSF) with a normal opening pressure, pleocytosis, elevated red blood cells, elevated IGGs, elevated protein count, and normal glucose levels. The CSF was positive for VZV DNA without fungal or bacterial growth (Table 3). Additional autoimmune, malignancy, and infectious workup were negative (Table 2).

**Table 1:** Laboratory Values From Serum CRP, C-reactive protein; ESR, erythrocyte sedimentation rate; INR, international normalized ratio; PT, prothrombin time; aPTT, activated partial thromboplastin time; TSH, thyroid-stimulating hormone; WBC, white blood cells; RBC, red blood cells; Hgb, hemoglobin; MCV, mean corpuscular volume.

Test	Patient Value	Reference Range
CRP (mg/dL)	0.06	0-0.5
ESR (mm/h)	73	0-15
INR	0.9	0.8-1
PT (s)	10.9	10.5-14.8
aPTT (s)	24.6	24-36.5
TSH (uIU/mL)	2.49	0.35-4.94
Absolute Lymphocyte Count (/uL)	383	1000-4800
% CD4	3	33-66
Absolute CD4 (/uL)	11	500-2600
WBC (10^3^/uL)	3.06	4.6-10.2
Total Lymphocytes (10^3^/uL)	0.58	0.69-4.79
RBC (10^6^/uL)	4.31	4.69-6.13
Hgb (g/dL)	12.6	14-18
MCV (fL)	92.6	80-97
Hematocrit (%)	39.9	43.5-53.7
Platelets (10^3^/uL)	178	142-424
Total Protein (g/dL)	8.4	6.4-8.3
Albumin (g/dL)	3.8	3.5-5
B12 (pg/mL)	254	213-816
Folate (ng/mL)	8.6	7-31
Vitamin D (ng/mL)	18.8	>30

**Table 2:** Non-Cerebrospinal Fluid Infectious Workup VZV, varicella-zoster virus; MRSA, methicillin-resistant *Staphylococcus aureus*; Ab, antibody; Ag, antigen; PCR, polymerase chain reaction; IgG, immunoglobulin G; IgM, immunoglobulin M.

Test	Patient Value	Reference Range
HIV RNA PCR Serum	311,458	<30 copies/mL
HIV 1 Ab Serum	Positive	Negative
HIV 2 Ab Serum	Negative	Negative
VZV IgG Serum	Positive	Negative
VZV DNA PCR Serum	Positive	Negative
VZV IgM Serum	Negative	Negative
MRSA DNA Nares	Negative	Negative
Chlamydia Urine PCR	Negative	Negative
Gonorrhea Urine PCR	Negative	Negative
Treponemal Ab Serum	Negative	Negative
Hepatitis A IgM Serum	Negative	Negative
Hepatitis B Surface Ag Serum	Negative	Negative
Hepatitis B Core IgM Serum	Negative	Negative
Hepatitis C Ab Serum	Negative	Negative
TB Quantiferon Serum	Negative	Negative
COVID-19 PCR	Negative	Negative
Cryptococcal Ag Serum	Negative	Negative
West Nile Virus Ab IgG Serum	Negative	Negative
West Nile Virus Ab IgM Serum	Negative	Negative
Oligoclonal Bands Serum	Negative	Negative

**Table 3:** Laboratory Parameters From Lumbar Puncture CSF Specimen Ab, antibody; Ag, antigen; MOG, myelin oligodendrocyte glycoprotein; CSF, cerebrospinal Fluid; VDRL, Venereal Disease Research Laboratory; EBV, Epstein-Barr virus; CMV, cytomegalovirus; HSV, herpes simplex virus; HHV, human herpesvirus; IgG, immunoglobulin G.

CSF Parameters	Patient Value	Reference Range
Color	Colorless	Colorless
Character	Clear	Clear
Nucleated Cells (/uL)	51	0-4
Red Blood Cells (/uL)	33	0-0
Lymphocytes (%)	77	40-80
Monocytes (%)	21	15-45
Eosinophils (%)	1	0-0
Neutrophils (%)	1	0-6
IgG (mg/dL)	10.35	0.93-5.86
Glucose (mg/dL)	66	40-70
Total Protein (mg/dL)	77	15.0-45.0
Myelin Basic Protein (mcg/L)	11.8	>4.0
MOG Ab	Negative	Negative
Oligoclonal Bands	Negative	Negative
VZV Zoster DNA	Positive	Negative
*Toxoplasma gondii* DNA	Negative	Negative
VDRL	Negative	Negative
EBV	Negative	Negative
HSV 1	Negative	Negative
HSV 2	Negative	Negative
Histoplasmosis Ag	Negative	Negative
Culture	No growth	No growth
Cryptococcal Ag	Negative	Negative
*Cryptococcus neoformans* GATII DNA	Negative	Negative
Flow Cytometry	No clonal B-cell population	No clonal B-cell population
Cytology	No malignant cells	No malignant cells
CMV DNA	Negative	Negative
*Escherichia coli* DNA	Negative	Negative
Enterovirus DNA	Negative	Negative
HHV6 DNA	Negative	Negative
*Haemophilus influenzae* DNA	Negative	Negative
*Listeria monocytogenes* DNA	Negative	Negative
*Neisseria meningitidis* DNA	Negative	Negative
Parechovirus RNA	Negative	Negative
*Streptococcus agalactiae* DNA	Negative	Negative
*Streptococcus pneumoniae* DNA	Negative	Negative

**Figure 1:**
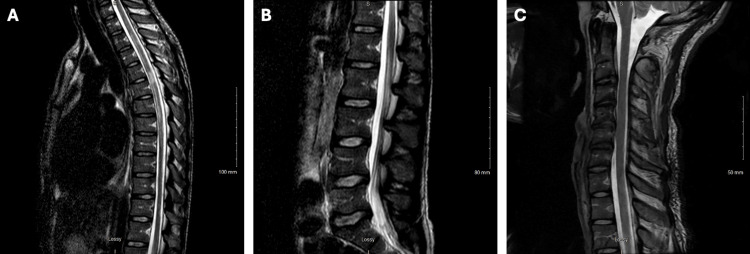
MR Imaging of Thoracic, Lumbar, and Cervical Spinal Cord in VZV Myelitis A: Sagittal T2-weighted sequence of the thoracic spinal cord with patchy hyperintense signal and mild enlargement of the cord, with the largest spinal cord hyperintensity at the T4-5 level involving most of the central cord. B: Sagittal T2-weighted sequence of the lumbar spinal cord with enhancement of cauda equina nerve roots and mild degenerative disc disease. C: Sagittal T2-weighted sequence of the cervical spinal cord with abnormal signaling visualized in the upper thoracic spinal cord extending superiorly into the cervical spinal cord at C7 level, no definite signal abnormality at C1-C6 levels, and mild degenerative disc disease. MR, magnetic resonance; VZV, varicella-zoster virus.

MR thoracic spine showed abnormal signal throughout the thoracic spinal cord, most prominent at T4-5, without masses or signs of compression (Figure 1A). MR lumbar spine demonstrated enhancement of cauda equina nerve roots throughout the lumbar spinal canal, mild degenerative disc disease, mild spinal canal stenosis at L4-L5, and mild to moderate neural foraminal stenosis bilaterally at L5-S1 (Figure 1B). MR cervical spine depicted an abnormal signal within the visualized upper thoracic spinal cord that extended superiorly into the cervical spinal cord to the C7 level (Figure 1C). Subsequent imaging of the MRI brain and CT head did not show acute abnormalities or demyelination.

The patient was diagnosed with thoracic demyelination, lumbosacral plexopathy, and arachnoiditis of cauda equina nerve roots secondary to VZV myelitis in the setting of HIV/AIDS. Due to acyclovir shortage, the team initiated ganciclovir 325 mg (5 mg/kg based on the total body weight of 63.5 kg) intravenous (IV) infusion every 12 hours on day 1. Given the existing literature on similar cases and the severity of his deficits with concern for long-term outcomes, he received dexamethasone 10 mg by mouth once, followed by dexamethasone 4 mg every 6 hours for two days starting on day 1. After ruling out cryptococcal meningitis on day 2, Infectious Disease (ID) initiated antiretroviral therapy with bictegravir-emtricitabine-tenofovir alafenamide 50-200-25 mg by mouth daily to reduce HIV viral load. ID also began *Pneumocystis jirovecii* Pneumonia prophylaxis with sulfamethoxazole-trimethoprim 800-160 mg by mouth daily. Additional treatment included cyanocobalamin 1000 mcg intramuscular injections weekly and duloxetine 30 mg by mouth twice daily for neuropathic pain. For constipation and urinary incontinence, a bowel regimen and bladder scans with intermittent catheterization were scheduled. For the debility and malnutrition, we consulted physical therapy, occupational therapy, and clinical nutrition. Following treatment initiation, the patient demonstrated an improvement in urinary incontinence, right lower-extremity sensation with return of nociception, and right plantarflexion to 2-3/5 strength. However, ID and Neurology were concerned about the immunosuppressive effects of corticosteroids with a CNS infection, which led to an initial discontinuation of corticosteroids on day 3. Following corticosteroid cessation, there was a worsening and plateau in symptom progression with decreased voluntary urination that required an indwelling bladder catheter and a decrease in plantarflexion strength to 1/5. Thus, on day 5, dexamethasone was resumed at 4 mg IV every 6 hours for four additional days, followed by oral prednisone taper at 25 mg twice a day, decreasing by 5 mg every three days.

After 10 days of ganciclovir, he was transitioned to valacyclovir 1 g by mouth three times a day and continued the planned oral prednisone taper and remaining treatment regimen listed above. He was discharged to inpatient rehabilitation and made mild functional progress with lower-extremity movement and bladder training. Repeat CSF and serum VZV PCR were negative two months following discharge. Unfortunately, the patient has required recurrent hospitalizations for myelitis complications such as bacteremia from urinary tract infection and debility.

## Discussion

While the pathogenesis of VZV myelitis is complex, the associated neuroinflammation likely involves a complex relationship between immune-mediated responses and viral replication within neuronal tissue, leading to tissue damage and dysfunction [1]. As seen in our patient, this destruction can be visualized best on T2-weighted and T2-weighted fluid-attenuated inversion recovery (FLAIR) magnetic resonance imaging of the spinal cord with focal or longitudinal serpiginous enhancing lesions [6]. Lumbar puncture will demonstrate CSF inflammation with pleocytosis, often neutrophilic, and raised protein concentrations [7]. A rapid search for VZV DNA through PCR or VZV IgG antibodies in CSF is essential for diagnosis. It is important to note that serum analysis for VZV antibodies has low clinical utility for diagnosing VZV myelitis, as many adults already have antibodies present in serum [8]. Furthermore, false-negative lab results can occur due to the timing of CSF sampling, as VZV DNA is more detectable in the first seven days, while antibodies are more detectable thereafter; thus, a negative result alone does not exclude VZV infection [9,10].

VZV myelitis is increasingly associated with immunosuppressed states as cell-mediated immunity to VZV wanes [11,12]. While prognosis is generally thought to be favorable in immunocompetent patients, with a complete recovery in up to 75% of cases, the outcome is much less optimistic in immunocompromised patients [3-5]. A literature review of VZV myelitis case reports between 1980 and 2012 demonstrated 31 cases in which most patients were immunocompromised, many with AIDS. These patients exhibited atypical presentations with poorer outcomes, including incomplete recovery and higher mortality relative to immunocompetent patients. Atypical presentations included myelopathy in the absence of skin lesions, as seen in our patient, which is likely due to decreased T-cell responsiveness [5]. There is even a significant disease burden of subclinical VZV infection in immunocompromised patients, as an investigation of CSF samples from HIV-positive individuals revealed anti-VZV intrathecal antibody synthesis in 16% of patients [13]. This highlights the importance of a heightened suspicion of VZV infections in these patient populations.

A large point of debate in this understudied condition is the standard of treatment, especially concerning the use of corticosteroids. Typically, antiviral therapy with IV acyclovir is used to inhibit viral DNA polymerase, with dosing ranging from 10 to 15 mg/kg every 8 hours [1]. Duration of IV acyclovir is often determined by the progression of skin lesions, which were absent in our patient’s presentation. In our patient, ID prescribed ganciclovir for systemic distribution with IV administration as acyclovir was not available due to shortage, and it has been considered an adequate alternative, especially in VZV meningoencephalitis [14]. Patients may then be transitioned to oral agents with adequate bioavailability, such as valaciclovir 1000 mg by mouth three times a day or famciclovir 500 mg by mouth three times a day [2]. While there is a paucity of data related to their use, the addition of corticosteroids has been recommended by several reports about VZV myelitis in immunocompromised patients, with potential benefits relating to known vascular inflammation in tissue samples from patients with CNS complications secondary to VZV infection [15]. There is no data on the specific timeframe for starting steroids about symptom onset. Even if steroids are initiated promptly, they do not guarantee resolution of neurological complications, as demonstrated in our case and other previously published cases [15]. There is a wide range of reported strategies in the limited available literature, including both oral and IV formulations, as well as a variety in dosing, duration, and decision to taper [4,15-17]. In other instances of VZV-induced neurologic dysfunction, like VZV vasculopathy, the use of IV corticosteroids is thought to be beneficial to the clinical course [18,19]. Our patient demonstrated an improvement in symptoms following dexamethasone initiation and apparently diminished progress following cessation. It is important to consider that symptom improvement with corticosteroids may have been confounded by time and/or additional medical therapy. Nonetheless, we believe that the utility of corticosteroids in these situations should at least be considered and may even outweigh the associated medication risks, like hypertension, bone fractures, or metabolic dysfunction [20]. Additional therapies that will also require future evaluation include intravenous immunoglobulin and plasmapheresis to regulate the immune response and lower inflammation [1].

## Conclusions

This case highlights the potential benefit of early corticosteroid therapy in the management of VZV myelitis, alongside antiviral treatment, to improve clinical outcomes. Our patient demonstrated notable neurological improvement following the initiation of a corticosteroid, emphasizing their possible role in modulating the inflammatory response associated with VZV reactivation. Despite current case reports supporting their use, standardized guidelines for corticosteroid administration in VZV myelitis remain lacking. This underscores the need for further research through larger studies to establish optimal treatment protocols and clarify the long-term impact of adjunctive corticosteroid therapy. Future investigations should also explore the pathophysiological mechanisms underlying VZV-induced inflammation to refine therapeutic strategies. By addressing these gaps, we can improve the management and prognosis of patients affected by this rare but serious condition.
